# Influence of Ripeness and Drying Process on the Polyphenols and Tocopherols of *Pistacia vera* L

**DOI:** 10.3390/molecules14114358

**Published:** 2009-10-30

**Authors:** Gabriele Ballistreri, Elena Arena, Biagio Fallico

**Affiliations:** University of Catania, DOFATA, Via S. Sofia 98, 95123 Catania, Italy; E-Mails: alegab@tiscalinet.it (G.B.); earena@unict.it (E.A.)

**Keywords:** anthocyanins, antioxidants, drying, flavonoids, stilbenes

## Abstract

This paper highlights, for the first time, the changes in the phenolics fraction (anthocyanins, flavonoids and stilbenes) and tocopherols of unpeeled *Pistacia vera* L. var. *bianca* with ripening, and the effect of the sun-drying process. The total polyphenol levels in pistachios, measured as mg of Gallic Acid Equivalent (GAE), were: 201 ± 10.1, 349 ± 18.3 and 184.7 ± 6.2 mg GAE/100 g DM in unripe, ripe and dried ripe samples, respectively. Most phenolics in ripe pistachios were found to be anthocyanins. They increased with ripening, while the sun drying process caused a susbtantial loss. Flavonoids found in all pistachio samples were daidzein, genistein, daidzin, quercetin, eriodictyol, luteolin, genistin and naringenin, which decreased both with ripening and drying. Before the drying process both unripe and ripe pistachios showed a higher content of *trans*-resveratrol than dried ripe samples. γ-Tocopherol was the major vitamin E isomer found in pistachios. The total content (of α- and γ-tocopherols) decreased, both during ripening and during the drying process. These results suggested that unpeeled pistachios can be considered an important source of phenolics, particularly of anthocyanins. Moreover, in order to preserve these healthy characteristics, new and more efficient drying processes should be adopted.

## Introduction

Clinical trials and epidemiological studies have established that increased consumption of nuts contributes to improved health and well being in humans [1]. This positive influence is attributed to the content of monounsaturated fats, vitamins and antioxidants, especially of phenolic compounds. These metabolites prevent oxidation of LDL cholesterol, a key step in atherogenesis [2]; moreover they promote the prevention of chronic diseases, such as cancer and cardiovascular diseases [3,4].

*Pistacia* species have attracted the attention of researchers for their antioxidant potential, besides their antimicrobial, anti-inflammatory and cytotoxic activities, particularly thanks to their flavonoid and other phenolic constituents [5,6,7]. Recently, the edible pistachio nut has been ranked among the top 50 food products with antioxidant potential [8]. The essential oils of the fruits and the leaves of pistachio, having antibacterial and antifungal properties, have been analyzed by GC and GC/MS [9].

*Pistacia vera* L., belonging to the *Anacardiaceae* family, is the only specie of the genus *Pistacia* producing edible nuts. The pistachio nut is mainly used in the confectionery industry and is consumed raw, sun-dried or roasted [10], while the shells have been proposed as a raw material to prepare activated carbons [11]. In the Mediterranean countries, the sun-drying process is the most used method to reduce the moisture level of pistachio nuts. The Italian pistachio, coming from Bronte (Southern Italy), which represents the 80% of the Italian production [12], shows, at ripeness, a red-violet skin while retaining a green nutmeat [13]. In other production areas, in order to have and sell a green product (a consumer preference), the picking time occurs some weeks in advance, at the end of August or by the beginning of September.

Pistachio kernels contain a remarkable amount of phenolic compounds such as anthocyanins, flavonoids, stilbenes, and low amount of antioxidant vitamins, but the quantity of such constituents is deeply influenced by the roasting [6] and bleaching processing [14] employed.

A survey of the evolution of the main bioactive components in pistachio kernels, during ripening could provide valuable information on the stability and antioxidative capacities of raw nuts before industrial processing. Lower levels of bioactive phenolics in pistachios may negatively impact the potential health benefits arising from pistachio consumption and favour lipid oxidation. Furthermore, polyphenols evolution during pistachio nut ripening has not been studied.

Following previous investigations on the Italian pistachio [13,15], we now report the results of a study on the evolution of phenolics component and tocopherols during ripening in *Pistacia vera* grown in Italy. In addition, the effect of the sun-drying process on these compounds was evaluated.

## Results and Discussion

The moisture of pistachios, the acidity and the peroxide number of the extracted oils were determined to verify the drying process capability as well as lipid oxidation rate (Table 1). The maturation process causes a dehydration of pistachio nuts [16], and unripe samples had a greater content of water (50.7%) than the ripe ones (35.3%). Moisture content in ripe pistachio kernels was reported to be generally around 37-40% [17]. Obviously, dried kernels had the lowest moisture content (3.3%).

The acidity mean values were about 0.3% for unripe pistachios and 0.5% for ripe ones, the peroxide number ranged from 0.6% to 1.0%, respectively; all pistachio samples had the same acidity and peroxide number according to literature [15]. This suggested that the sun-drying process did not have detrimental effect on the oxidative stability of pistachios confirming the results in [18].

**Table 1:** Moisture of pistachio kernels, acidity and peroxide number of *Pistacia vera* oils.

	Unripe kernels	Ripe kernels	Dried ripe kernels
**Moisture (%)**	50.7 ± 3.1^c^	35.3 ± 3.2^b^	3.3 ± 0.3^a^
**Acidity (%)**	0.3 ± 0.1^a^	0.5 ± 0.1^a^	0.5 ± 0.1^a^
**Peroxide number (meq/kg)**	0.6 ± 0.2^a^	1.0 ± 0.2^a^	1.0 ± 0.3^a^

Values are the mean ± SD (*n* = 4 per group); In the rows, values with different letters (a-c) are statistically different (P-Value < 0.05).

Total Polyphenols (TP), as described in the Experimental section, were determined by the Folin Ciocalteau method. It is well known that acidified methanol (contaning hydrochloric, acetic or formic acid) is the best solvent for the extraction of anthocyanins [14,19,20], while non-quantitative recovery of anthocyanins is possible using pure methanol. 

Preliminarily, in order to establish the best order of extracting solvents, a set of trials were carried out. When the order was: acidified methanol then pure methanol, the extraction was not selective. In fact, the first fraction extracted all the anthocyanins plus other phenolics. When the order was the opposite (methanol followed by acidified methanol) the extractions were much more selective (data not shown). Moreover, in order to avoid an overestimation of the TP level of pistachios, the two extractions were carried out on the same aliquot of pistachio powder. 

**Table 2:** Mean content of total phenols (mg of GAE/100 g DM) in *Pistacia vera* nuts.

Samples	Methanol extract	Acidic Methanol extract	Combined extracts
Unripe	185.2 ± 5.1^c^	23.2 ± 2.3^a^	201 ± 10.1^b^
Ripe	150.9 ± 12.4^b^	202.1 ± 13.8^c^	349 ± 18.3^c^
Dried ripe	107.3 ± 1.5^a^	81.7 ± 1.9^b^	184.71 ± 6.2^a^

Values are the mean ± SD (*n* = 4 per group); In the columns, values with different letters (a-c) are statistically different (P-Value < 0.05).

Table 2 reports the content of TP determined in methanolic, methanolic acidic and in the combined methanolic extracts of the pistachio samples. The TP level of methanolic extract of unripe samples was higher than in ripe ones, with an average value of 185.2 ± 5.1 and 150.9 ± 12.4 mg/100 g DM, respectively. After the drying process, the average polyphenols content in ripe samples decreased up to 107.3 ± 1.5 mg/100 g DM (Table 2). This reduction (-42%) can be ascribed both to the ripening (-18.5%) and to the sun-drying process (-23.6%). Such an effect can be attributed to a phenolics reduction (Table 3).

The TP values of acidic methanolic extracts of unripe pistachios were the lowest, with an average content of 23.2 ± 2.3 mg/100 g, DM, which increased up to 202.1 ± 13.8 mg/100 g DM at ripeness, decreasing up to 81.7 ± 1.9 mg/100 g DM after the sun-drying process (Table 2). This can be explained by the contribution of anthocyanins. These pigments are localized exclusively in pistachios’ skin [13,14] and their level increases, as in flowers and other fruits, with ripening, but they are easily destroyed by heating [14,21].

The differences between the Total Polyphenols content of the methanolic extracts and the acidified methanolic ones could be explained by the phenolics extracted. There was a very high correlation (*R*^2^ = 0.9722) between total polyphenols of the methanol extract and the total flavonoids amount and between the total polyphenols of acidic methanolic extracts and the total anthocyanins amount (*R*^2^ = 0.9997).

Data in Table 2 show that the TP levels of the combined extracts of unripe, ripe and dried pistachios were almost the same to the sum of total polyphenols determined in methanol and acidic methanol extracts, respectively. Ripening led to an increase of phenolics attributed to anthocyanins biosynthesis. In fact, the unripe samples had a lower level of total phenolics, 201 ± 10.1 mg GAE/100 g DM, respect to the ripe samples, 349 ± 18.3 mg GAE/100 g DM. The sun-drying process induced a deep decrease (-47%) to 184.7 ± 6.2 mg GAE/100g DM. This behavior reflects the flavonoids and anthocyanins loss with the drying process (Table 3). These values are lower than those reported in the literature [22]. 

**Table 3:** Content of Phenolics (mg/100 g DM) in edible kernels of Pistacia vera.

Phenolics	Unripe kernels	Ripe kernels	Dried ripe kernels
*Anthocyanins*			
cyanidin-3-galactoside	4.5 ± 0.2^a^	48.6 ± 4.2^c^	20.4 ± 1.0^b^
cyanidin-3-glucoside	2.4 ± 0.6^a^	15.1 ± 1.2^c^	3.9 ± 2.9^ab^
Total	6.9	63.7	24.3
*Flavonoids*			
Daidzein	5.2 ± 0.5^c^	3.3 ± 0.6^b^	2.1 ± 0.1^a^
Genistein	5.0 ± 0.4^c^	3.2 ± 0.3^b^	2.0 ± 0.1^a^
Daidzin	2.5 ± 0.2^c^	1.7 ± 0.2^b^	1.2 ± 0.1^a^
Quercetin	2.3 ± 0.1^c^	1.7 ± 0.3^b^	1.4 ± 0.1^ab^
Eriodictyol	2.1 ± 0.3^c^	1.4 ± 0.2^b^	0.9 ± 0.2^ab^
Luteolin	2.1 ± 0.3^c^	1.4 ± 0.2^b^	0.9 ± 0.2^ab^
Genistin	1.9 ± 0.5^b^	1.1 ± 0.1^a^	1.1 ± 0.3^a^
Naringenin	0.3 ± 0.1^c^	0.2 ± 0.1^b^	0.1 ± 0.0^ab^
Total	21.5	14.0	9.6
*Stilbenes*			
*trans-*resveratrol	1.7 ± 0.1^c^	1.2 ± 0.1^b^	0.2 ± 0.1^a^

Values are the mean ± SD (*n* = 4 per group); In the rows, values with different letters (a-c) are statistically different (P-Value < 0.05).

Table 3 shows the levels of identified phenolics extracted from pistachios. The major compounds in ripe samples were anthocyanins (cyanidin-3-galactoside and cyanidin-3-glucoside). The sum of the two anthocyanins in ripe samples (63.7 mg/100 g) was tenfold higher than the unripe ones (Table 3). These data confirm that the high TP content of acidic methanol extracts in ripe products (Table 2) is due to a very high level of anthocyanins. Cyanidin-3-galactoside increased its concentration, with ripening, faster than cyanidin-3-glucoside (Table 3). 

The sun-drying process caused the loss of about the 60% of anthocyanins. Cyanidin-3-glucoside seems to be more sensitive to the drying process than cyanidin-3-galactoside. In fact, the ratio between cyanidin-3-galactoside and cyanidin-3-glucoside was 3.22 in raw ripe pistachios and 5.23 after the sun-drying process, respectively (Table 3). Similar ratio values, 3.3 for raw and 5.31 for roasted samples, respectively, can be extrapolated from the data reported by [14]. Such a ratio (cy-3-gal/cy-3-glu) could be a useful index to evaluate thermal treatments of pistachios, but, further investigations are needed. 

Pistachios have always been valued for their sensorial properties and recent studies confirm that they are a good source of antioxidants [8]. To our knowledge, they have never been considered as a meaningful source of anthocyanins. The anthocyanin levels in ripe Italian pistachios (64 mg/100 g DM) are tenfold higher than those found in blood orange juice (about 7 mg/100 mL) [23], and between 50-76% of the anthocyanin level found in unpasteurized and pasteurized blackberry juice, respectively (about 130 and 84 mg/100 mL) [24]. Both of these are considered to be very good sources of anthocyanins. Although after the sun-drying process there is a loss of about 60% of anthocyanins, their level is still significant (about 24 mg/100 g DM). Considering that the serving size of pistachios is 28.35 g [8], this would mean a daily anthocyanins intake from ripe or dried ripe pistachios of 19 or 7 mg, respectively. Obviously the intake of pistachio phenolics is related to the consumption of unpeeled product, as these substances are contained on the nuts skin [14].

Table 3 also reports the level of the eight identified flavonoids: daidzein, genistein and their glycoside derivatives daidzin and genistin, eriodictyol, quercetin, luteolin and naringenin. The major flavonoids in all samples were daidzein and genistein; they represent the 50% of total flavonoids. For the first time on pistachios were detected the isoflavone-glucosides daidzin and genistin. In soybeans isoflavone-glucosides are predominant respect to the aglycons [25,26] while the contrary is true in pistachios. In previous studies the presence of biochanin A [7], glycitein and formononetin has been reported, although at very low levels [7,27]. These isoflavones were not detected in our pistachio samples. The high variability of isoflavones was reported both in pistachios [6,7,14,27] and in other foods. In fact, the isoflavones content depends on variety, environmental factors, growth, harvesting, processing, sampling and analytical method [7,28].

Total flavonoids content decreased from about 21.5 mg/100 g DM to 14.0 mg/100 g DM with ripening. It induces a reduction of about 30-36% of each flavonoid, with the exception of genistin (-42%) and quercetin (-26%). The behavior of isoflavones was the opposite respect to that highlighted by [26] during soybeans ripening. 

The stability of flavonoids to the sun-drying process was different, in fact naringenin seems to be the more sensitive with a loss of 50%, daidzein, genistein, eriodictyol and luteolin recorded a loss of about 36%, quercetin of about 22%, while genistin was the most stable flavonoid and no loss was detected after the drying process.

The phytoalexin resveratrol (3,5,4'-trihydroxystilbene) is an important bioactive polyphenol [29]. Stilbenes act against biotic and abiotic factors, defending plants from cold, heat, fungal infections and the growth of molds. Resveratrol has been detected in several plant species such as grapes (*Vitis vinifera*) [30], edible peanuts (*Arachis hypogaea* L.) and pistachios cultivars grown in Turkey [31].

The *trans*-resveratrol concentration ranged from 1.7 mg/100 g DM in unripe kernels to 1.2 mg/100 g DM in ripe kernels. While the drying process induced a profound loss (about 84%) of this compound, down to 0.2 mg/100 g DM (Table 3). The immature kernels had greater capacity to produce phytoalexin than mature ones [31,32,33]. Taking into account that red wines contain from 0.1 up to 14.3 mg/L of *trans*-resveratrol [34], both unripe and ripe pistachios can be considered a good source of stilbenes. The level of *trans*-resveratrol in dried ripe pistachios (Table 3) was higher than those reported in literature, both for the Italianproduct [6,35] and for Turkish pistachios [31].

Table 4 shows that γ-tocopherol is the major vitamin E isomer found in all pistachio samples. This is in agreement with literature data [6,22]. The tocopherols concentration ranged from 16.9 mg/100 g DM in unripe kernels up to 13.5 mg/100 g DM in ripe ones, the dried samples showed a loss of about 38% of these vitamins. γ-Tocopherol has higher antioxidant activity compared to α-tocopherol, better preserving the lipidic fraction of pistachio from oxidation [36]. 

**Table 4:** Content of Tocopherols (mg/100 g DM) in edible kernels of Pistacia vera.

Tocopherols	Unripe kernels	Ripe kernels	Dried ripe kernels
α-tocopherol	0.7 ± 0.1^c^	0.6 ± 0.0^abc^	0.4 ± 0.1^a^
γ-tocopherol	16.2 ± 0.9^c^	12.9 ± 1.1^b^	8.0 ± 0.5^a^
Total	16.9	13.5	8.4

Values are the mean ± SD (*n* = 4 per group); In the rows, values with different letters (a-c) are statistically different (P-Value < 0.05).

The Total Antioxidant Activity (TAA) was calculated as Trolox Equivalent Antioxidant Capacity (TEAC) value x C_mmol/kg_, (Table 5) as reported for other fruits [23,37,38].

**Table 5:** Calculated TAA of Phenolics and tocopherols in edible kernels of *Pistacia vera*.

		Unripe kernels		Ripe kernels		Dried Ripe kernels	
Phenolics	TEAC	mmol/kg	TAA calcd	mmol/kg	TAA calcd	mmol/kg	TAA calcd
***Anthocyanins***							
cyanidin-3-galactoside	2.9^a^	0.10	0.29	1.08	3.13	0.45	1.32
cyanidin-3-glucoside	2.47^b^	0.05	0.13	0.34	0.83	0.09	0.21
Total^1^		0.15	0.42	1.42	3.96	0.54	1.53
***Flavonoids***							
Daidzein	1.2^c^	0.21	0.25	0.13	0.16	0.08	0.10
Genistein	1.0^c^	0.18	0.18	0.12	0.12	0.07	0.07
Daidzin	-	0.06	nc	0.04	nc	0.03	Nc
Quercetin^a^	4.7^a^	0.08	0.36	0.06	0.27	0.04	0.21
Eriodictyol	-	0.07	nc	0.05	nc	0.03	nc
Luteolin^a^	2.1^a^	0.07	0.16	0.05	0.10	0.03	0.06
Genistin	-	0.04	nc	0.03	nc	0.03	nc
Naringenin^a^	1.5^a^	0.01	0.02	0.01	0.01	0.00	0.01
Total^2^		0.72	0.97	0.49	0.66	0.31	0.46
*****Stilbenes*****							
*Trans-*resveratrol^ 3^	2.7^a^	0.08	0.21	0.05	0.15	0.01	0.02
*****Tocopherols*****							
α-tocopherol	1.0^d^	0.02	0.02	0.01	0.01	0.01	0.01
γ-tocopherol	1.0^d^	0.39	0.39	0.31	0.31	0.19	0.19
Total^4^		0.41	0.41	0.32	0.32	0.20	0.20
**TOTAL ^1+2+3+4^**		1.36	2.01	2.28	5.09	1.06	2.21

TEAC value reported by: ^a ^ [37]; ^b^ [38]; ^c^ [39]; ^d^ [40]. TAA calculated (TEAC valuexC_mmol/kg_); nc, not calculated.

The contribute of anthocyanins to TAA value was the highest (3.96) in ripe kernels, followed by dried samples (1.53) and unripe ones (0.42). The TAA values of flavonoids and *trans*-resveratrol decreased during ripening, from 0.97 to 0.66 and from 0.21 to 0.15, respectively. The sun-drying process reduced the TAA of anthocyanins of about 61%, of flavonoids of about 28%, of *trans*-resveratrol of about 85% and of tocopherols of about 48%. The main contribute to total TAA in unripe pistachios is due to flavonoids, while in ripe and dried samples is due to anthocyanins.

## Experimental

### Sampling

Twelve pistachio samples (5 kg each), grown in Bronte var. *bianca* (Catania, Italy), were collected as follows:

(1) From four different orchards, selecting five different trees in each one.
(2) Each at two different ripening stages: unripe (the 3^rd^ week of August) and ripe (2^nd^ week of September); the maturation stage was determined evaluating the nuts skin color [13] (*n* = 8).
(3) The ripe pistachios in shell were sun-dried for 3 days, to a moisture content of 3% (*n* = 4).

All samples were produced and collected in 2007, stored at 4 °C in darkness, at least up to 48 hours before analyses. The following analytical determinations were conducted on the pistachio samples: Acidity and peroxide number on oil, moisture, total polyphenols, flavonoids, anthocyanins, *trans*-resveratrol and tocopherols on pistachio powder.

### Reagents and standards

Absolute ethanol, acetic acid,acetonitrile and methanol were purchased from Lab Scan (Dublin, Ireland). Dichloromethane, hydrochloric acid, *n*-hexane, petroleum ether and water from JT. Baker (Deventer, Holland). All the reagents used were HPLC purity grade. The standards used for the studies were purchased from the Sigma-Aldrich Chemical Company (St. Louis, MO, USA): biochanin A, cyanidin-3-galactoside, cyanidin-3-glucoside, daidzein, daidzin, eriodictyol, formononetin, genistein, genistin, luteolin, naringenin, quercetin, *trans*-resveratrol, α-tocopherol, and γ-tocopherol. Glycitein was from Wako Pure Chemical Industries, Ltd (Osaka, Japan).

### Oil extraction and determination of oxidative parameters

For each sample 500 g of pistachio kernels were shelled, and the obtained unpeeled seeds were crushed in a home grinder (La Moulinette, Moulinex, 2002); subsequently pistachio powder (30 g) was mixed with *n*-hexane (50 mL) and stirred for 30 minutes to extract the oil. The *n*-hexane extract was filtered and the solvent was evaporated under reduced pressure, using a rotavapor (RE 111, Büchi, Switzerland). Acidity (%) and peroxide number were determined on pistachio oils according to standard methods [41]. All the analyses were conducted in triplicate.

### Moisture determination

Moisture content was determined on pistachio powder obtained after crushing kernels in a home grinder. Portions of fresh powder were used in triplicate to assess moisture content. They were placed in an oven at 105°C until dry weight was constant.

### Extraction and determination of total polyphenols

Polyphenols were extracted from 10 g of unpeeled pistachio powder with three aliquots of methanol (25 mL each for 10 minutes) in an ultrasonic bath (UST 6/2 SRV, Uesseti) in the dark. The methanolic extract was recovered. Then, in order to extract anthocyanins, the same pistachio powder was re-extracted with three aliquots of acidic methanol (HCl 0.1%) as described above. Total polyphenols were determined by Folin-Ciocalteu method [42] on the methanolic, the acidic methanolic and the combined methanolic extracts, respectively. The specific absorbance of the solution was measured at 760 nm using a spectrophotometer (Shimadzu UV 2401). The content of total polyphenols was calculated as ppm gallic acid equivalent (GAE), and was expressed as mg of GAE/100g of pistachio dry matter (DM). All the extractions were conducted in triplicate.

### Extraction and HPLC analysis of phenolics and tocopherols

The chromatographic apparatus was a Spectra System constituted of a P4000 pump with a quaternary gradient pump system, a SCM1000 vacuum membrane degasser, an UV6000LP diode-array detector, an AS3000 autosampler with a thermostated column compartment (Thermo Electron Corporation, Waltham, MA, USA). The column was a Phenomenex Luna C_18_ (250 mm × 4.6 mm, 5 µm). The HPLC was connected to Chromquest Chromatography Manager 4.2 (Rev.A.97202) software (Thermo Electron, San Jose, CA, USA) for determination of peaks areas. All HPLC analyses were carried out in triplicate. Anthocyanins were extracted from 10 g of unpeeled pistachio powder with three aliquots of acidic methanol (25 mL each for 10 minutes) in an ultrasonic bath (UST 6/2 SRV, Uesseti) in the dark. The same procedure, applied on different aliquots of samples, was used to extract flavonoids and *trans*-resveratrol with methanol. The extracts were concentrated by rotary evaporator under vacuum at 20 °C up to a small volume, diluted to a final volume of 5 mL with acidic methanol or methanol, then filtered (0.45 μm) before the HPLC injection.

Evaluation of flavonoids (daidzin, daidzein, genistin, genistein, eriodictyol, luteolin, naringenin, and quercetin) was performed by reversed-phase HPLC as reported in [6], with slight changes. Eluent A was 0.1% (v/v) acetic acid in water and eluent B was methanol. Solvent B was increased from 35 to 70% in 35 min, then from 70% to 90% in 3 min and finally from 90% to 10% in 10 min, at a constant flow rate of 1 mL/min. The injection volume was 50 μL. Spectra were recorded from 200 to 450 nm and monitored at 260 nm. For stilbenes (*trans*-resveratrol) and anthocyanins (cyanidin-3-galactoside; cyanidin-3-glucoside) the mobile phases and the gradient programs were according to [31] and [13], respectively. Tocopherols (α and γ-tocopherol) were extracted in triplicate and analyzed according to [6]. All the compounds were identified by comparing retention times and UV spectra with those of standards and by splitting each sample with standards. Quantification of each component was performed using calibration curves of external standards.

### Statistical analysis

Differences in the mean values of the variables reported in tables 1-4 were tested by analysis of variance (ANOVA), and the significance of differences between samples was determined using the F-test. The levels of significance were P *<* 0.05 at the 95% confidence level. Statistical analysis was performed using the Statgraphic Plus 4.1 software (Manugistic Inc. Rockville, MD, USA).

## Conclusions

Data shown above highlighted a loss of flavonoids, *trans*-resveratrol and tocopherols during ripening of pistachios, while total polyphenols increased. The sun drying process causes a profund loss of compounds with high antioxidant activity such as anthocyanins and *trans*-resveratrol (60 and 84%, respectively). The reduction of bioactive phenolics may affect the potential health benefits of pistachio consumption. Phenolics and tocopherols protect the nut fatty acids from oxidation. The main products of oxidation are hydroperoxides that are susceptible to further degradation processes by enzymatic action and physical or chemical factors. The unpeeled product, above all for its high anthocyanins content, can be considered as a good source of antioxidants, and may have a health-promoting potential offering protection against oxidative stress. A less detrimental pistachio drying processes would be very useful, both for health aspects, and to preserve their nutritional values.

## References

[1] Willet W.C. (2001). Eat, Drink, and be Healthy−The Harvard Medical School Guide to Healthy Eating.

[2] Chen C.Y., Milbury P.E., Lapsley K., Blumberg J.B. (2005). Flavonoids from almond skins are bioavailable and act synergistically with vitamin C and E to enhance hamster and human LDL resistance to oxidation. J. Nutr..

[3] Morton L.W., Caccetta Rima A.C., Puddey I.B., Croft K.D. (2000). Chemistry and biological effects of dietary phenolic compounds: relevance to cardiovascular disease. Clin. Exp. Pharmacol. Physiol..

[4] Kris-Etherton P.M., Hecker K.D., Bonanome A., Coval S.M., Binkoski A.E., Hilpert K.F., Griel A.E., Etherton T.D. (2002). Bioactive compounds in foods: Their role in the prevention of cardiovascular disease and cancer. Am. J. Med. Sci..

[5] Topcu G., Ay M., Bilici A., Sarikurkcu C., Ozturk M., Ulubelen A. (2007). A new flavone from antioxidant extracts of *Pistacia terebinthus*. Food Chem..

[6] Gentile C., Tesoriere L., Butera D., Fazzari M., Monastero M., Allegra M., Livrea M.A. (2007). Antioxidant Activity of Sicilian Pistachio (*Pistacia vera* L. Var. Bronte) Nut extract and Its Bioactive Components. J. Agric. Food Chem..

[7] Kuhnle G.G.C., Dell’Aquila C., Aspinall S.M., Runswick S.A., Mulligan A.A., Bingham S.A. (2008). Phytoestrogen content of beverages, nuts, seeds, and oils. J. Agric. Food Chem..

[8] Halvorsen B.L., Carlsen M.H., Phillips K.M., Bohn S.K., Holte K., Jacobs D.R., Blomhoff R. (2006). Content of redox-active compounds (i.e., antioxidants) in foods consumed in the United States. Am. J. Clin. Nutr..

[9] Tsokou A., Georgopoulou K., Melliou E., Magiatis P., Tsitsa E. (2007). Composition and enantiomeric analysis of the essential oil of the fruits and the leaves of *Pistacia vera* from Greece. Molecules.

[10] Angelini P. (1987). I mercati mondiali del pistacchio. Agricol. Ricer. II.

[11] Galvano F., Pietri A., Fallico B., Bertuzzi T., Scirè S., Galvano M., Maggiore R. (1996). Activated carbons: in vitro affinity for aflatoxin B1 and relation of absorption ability to physicochemical parameters. J. Food Protect..

[12] Bellia F. (2007). Indagini territoriali ed aziendali sulla frutticoltura etnea: Risultati economici e prospettive. Principali risultati del Progetto di Ricerca Miglioramento e Valorizzazione delle Produzioni Frutticole Etnee.

[13] Bellomo M.G., Fallico B. (2007). Anthocyanins, chlorophylls and xantophylls in pistachio nuts (*Pistacia vera*) of different geographic origin. J. Food Compos. Anal..

[14] Seeram N.P., Zhang Y., Henning S.M., Lee R., Niu Y., Lin G., Heber D. (2006). Pistachio skin phenolics are destroyed by bleaching resulting in reduced antioxidative capacities. J. Agric. Food Chem..

[15] Arena E., Campisi S., Fallico B., Maccarone E. (2007). Distribution of fatty acids and phytosterols as a criterion to discriminate geographic origin of pistachio seeds. Food Chem..

[16] Chahed T., Hamrouni I., Dhifi W., Msaada K., Kchouk M.E., Marzouk B. (2006). Lipid evaluation during the development of pistacchio seed from the region of *Kairouan* (middle of Tunisia). J. Food Lipids.

[17] Kashaninejad M., Mortazavi A., Safekordi A., Tabil L.G. (2004). Some physical properties of pistachio (*Pistacia vera* L.) nut and its kernel. J. Food Eng..

[18] Maskam M., Karatas S. (1998). Fatty acid oxidation of pistachio nuts stored under variation atmospheric conditions and different temperatures. J. Sci. Food Agric..

[19] Gao L., Mazza G. (1994). Quantitation and distribution of simple and acylated anthocyanins and other phenolics in blueberries. J. Food Sci..

[20] Nicolosi Asmundo C., Arena E., Grasso A. (2007). Anthocyanic profile of different red grape cultivars grown in Sicily. Acta Horticult..

[21] Seeram N.P., Bourquin L.D., Nair M.G. (2001). Degradation products of cyaniding glycosides from tart cherries and their bioactivities. J. Agric. Food Chem..

[22] Kornsteiner M., Wagner K.H., Elmadfa I. (2006). Tocopherols and total phenolics in 10 different nut types. Food Chem..

[23] Arena E., Fallico B., Maccarone E. (2001). Evaluation of antioxidant capacity of blood orange juices as influenced by constituents, concentration process and storage. Food Chem..

[24] Hager T.J., Howard L.R., Prior R.L. (2008). Processing and storage effects on monomeric anthocyanins, percent polymeric color, and antioxidant capacity of processed blackberry products. J. Agric. Food Chem..

[25] Wang H., Murphy P.A. (1994). Isoflavone content in commercial soybeans foods. J. Agric. Food Chem..

[26] Kumar V., Rani A., Kumar Dixit A., Bhatnagar D., Chauhan G.S. (2009). Relative Changes in Tocopherols, Isoflavones, Total Phenolic Content, and Antioxidative Activity in Soybean Seeds at Different Reproductive Stages. J. Agric. Food Chem..

[27] Thompson L.U., Boucher B.A., Liu Z., Cotterchio M., Kreiger N. (2006). Phytoestrogen content of foods consumed in Canada, including isoflavones, lignans and coumestan. Nutr. Canc..

[28] Kuhnle G.G.C., Dell’Aquila C., Runswick S.A., Bingham S.A. (2009). Variability of phytoestrogen content in foods from different sources. Food Chem..

[29] Bowers J.L., Tyulmenkov V.V., Jernigan S.C., Klinge C.M. (2000). Resveratrol acts as a mixed against/antagonist for estrogen receptors α and β. Endocrinology.

[30] Lamuela-Raventos R.M., Romero-Perez A.I., Waterhouse A.L., de la Torre-Boronat M.C. (1995). Direct HPLC analysis of cis and trans-resveratrol and piceid isomers in Spanish red *Vitis vinifera* wines. J. Agric. Food Chem..

[31] Tokusoglu O., Unal M.K., Yemis F. (2005). Determination of the Phytoalexin Resveratrol (3,5,4’-Trihydroxystilbene) in Peanuts and Pistachios by High-Performance Liquid Chromatographic Diode Array (HPLC-DAD) and Gas Chromatography-Mass Spectrometry (GC-MS). J. Agric. Food Chem..

[32] Dorner J.W., Cole R.J., Sanders T.H., Blankeship P.D. (1989). Interrelationship of kernel water activity, soil temperature, maturity and phytoalexin production in preharvest aflatoxin contamination of drought-stressed peanuts. Mycopathologia.

[33] Sanders T.H., McMichael R.W., Hendrix K.W. (2000). Occurence of resveratrol in edile peanuts. J. Agric. Food Chem..

[34] Baur J.A., Sinclair D.A. (2006). Therapeutic potential of resveratrol: the in vivo evidence. Nat. Rev. Drug Discov..

[35] Grippi F., Crosta L., Aiello G., Tolomeo M., Oliveri F., Gebbia N., Curione A. (2008). Determination of stilbenes in Sicilian pistachio by high-performance liquid chromatographic diode array (HPLC-DAD/FLD) and evaluation of eventually mycotoxin contamination. Food Chem..

[36] Belitz H.D., Grosch W., Schieberele P. (2004). Lipids. Food Chemistry.

[37] Rice-Evans C.A., Miller N.J., Paganda G. (1996). Structure-Antioxidant activity relationships of flavonoids and phenolic acids. Free Radic. Biol. Med..

[38] Miller N., Rice-Evans C.A. (1997). The relative contributions of ascorbic acid and phenolic antioxidants to the total antioxidant activity of orange and apple fruit juices and blackcurrant drink. Food Chem..

[39] Hundhausen C., Bosch-Saadatmandi C., Augustin K., Blank R., Wolffram S., Rimbach G. (2005). Effect of vitamin E and polyphenols on ochratoxin A-induced cytotoxicity in liver (HepG2) cells. J. Plant Physiol..

[40] Ruiz-Larrea M.B., Martìn C., Martìnez R., Navarro R., Lacort M., Miller N.J. (2000). Antioxidant activities of estrogens against aqueous and lipophilic radicals; differences between phenol and catecol estrogens. Chem. Phys. Lipids.

[41] (1991). Commission Regulation (EEC) No 2568/91 of 11 July 1991 on the characteristics of olive oil and olive-residue oil and on the relevant methods of analysis. Official Journal of the European Communities.

[42] Singleton V.L., Rossi A. (1965). Colorimetry of total phenolics with phosphomolybdic-phosphotungstic acid reagents. Am. J. Enol. Vitic..

